# Medicinal Plants to Strengthen Immunity during a Pandemic

**DOI:** 10.3390/ph13100313

**Published:** 2020-10-15

**Authors:** Olga Babich, Stanislav Sukhikh, Alexander Prosekov, Lyudmila Asyakina, Svetlana Ivanova

**Affiliations:** 1 Institute of Living Systems, Immanuel Kant Baltic Federal University, A. Nevskogo Street 14, 236016 Kaliningrad, Russia; olich.43@mail.ru (O.B.); stas-asp@mail.ru (S.S.); 2 Laboratory of Biocatalysis, Kemerovo State University, Krasnaya Street 6, 650043 Kemerovo, Russia; a.prosekov@inbox.ru; 3 Department of Bionanotechnology, Kemerovo State University, Krasnaya Street 6, 650043 Kemerovo, Russia; alk_kem@mail.ru; 4 Natural Nutraceutical Biotesting Laboratory, Kemerovo State University, Krasnaya Street 6, 650043 Kemerovo, Russia; 5 Department of General Mathematics and Informatics, Kemerovo State University, Krasnaya Street, 6, 650043 Kemerovo, Russia

**Keywords:** medicinal plants, antiviral activity, prevention, phenolic compounds, flavonoids

## Abstract

The development of new effective anti-coronavirus drugs and therapies is important, but it requires significant human, financial and, most importantly, time expenditures. The current pandemic is neither the first nor the last. Humanity has already accumulated considerable survival experience. We cannot do without prevention and epidemiological protection measures. This study reviews medicinal plants that grow in Northeast Asia and whose antioxidant, antiviral, anti-inflammatory and immunomodulatory characteristics are already known, also in the framework of the prevention and treatment of pneumonia of various etiologies. The need for a comprehensive approach to maintaining immunodefences, including functional foods and positive emotions, is emphasized. In the period of pandemics, it is important to research various areas that allow to us accumulate a critical mass of information and cope with the next global disease.

## 1. Introduction

Throughout history, mankind has been accompanied by infectious diseases that have, in one way or another, raised the question of its survival. This was the case with the Spanish influenza (H_1_N_1_ virus) at the beginning of the last century, which resulted in the death of 5% of the world’s population. Almost all the time, mutations in strains of influenza A viruses lead to the emergence of infectious diseases with new symptoms and consequences. Avian flu, swine flu and other zoonotic influenza virus infections in humans lead to diseases ranging from mild upper respiratory tract infections to severe pneumonia, acute respiratory failure syndrome and death [1]. Each of them is initially regarded as a pandemic, but as soon as a treatment medication and a vaccine are developed, it is considered a regular seasonal flu. The coronavirus that led to the COVID-19 pandemic is similar to the pathogen SARS-CoV (viral respiratory disease of zoonotic origin) that caused the epidemic of 2003. A drug for atypical pneumonia that has passed clinical trials is yet to be developed. In 2020, humanity is being forced to return to the unfinished solution of the problem, whose initial conditions will be amended with new criteria. This SARS-CoV-2 virus has affected many people, not only in China but spreading to almost all countries and territories in a short time [2,3]. Many countries (China, the United States, Germany, Great Britain, Russia, etc.) are intensively working on creating a vaccine, but even in this case, time is needed. The population only needs to wait for the work to be completed. However, every new day brings thousands of infected people [4], and some of them are not going to make it. Until there is a vaccine, all countries issue the same recommendations as follows: compliance with sanitary and hygienic standards; limited contacts up to complete self-isolation; strengthening of body’s defense systems that will both protect and lead to recovery in the event of infection [5,6].

Herbs are traditionally used in many therapeutic practices, if not as the main, then as the accompanying therapy in combination with medications, aimed at boosting immunity for prevention. Phytotherapy has repeatedly proven its effectiveness, including its ability to cope with infectious diseases [7]. There are medicinal plants whose extracts have an inhibitory effect on viruses such as herpes simplex virus type 2, HIV, hepatitis B virus (HBV), smallpox virus and severe acute respiratory syndrome, as well as on viral strains resistant to conventional antiviral drugs [8,9].

Plants from traditional Chinese medicine are rich sources of compounds used for the development of medicines for a wide range of diseases (from coughs and colds to parasitic infections and inflammations) [10,11,12]. Chinese scientists were the first to search for medicinal plants against a new coronavirus infection. On the one hand, they have centuries of experience in using medicinal plants both in treatment and prevention; on the other hand, the epicenter of COVID-19 is located on their territory [4,13].

The authors [7] proposed two principles for selecting medicinal plants (efficacy of oral administration and compatibility of traditional use). As a result, 26 plants were identified, of which only one is recommended for prevention—*Fortunes bossfern* (rhizome)—and the rest are recommended for intake at different stages of COVID-19 therapy.

It is believed that the most beneficial for a person are those fruits and plants that grow on the territory of their residence. On the other hand, the territories of China and the Far East are geographically close, which allows that preventive medicinal plants can be found on the territory of Northeast Asian. This review examines in vitro and in vivo studies that report on the immunomodulatory potential of medicinal plants growing in Northeast Asia.

## 2. Methods

Conducted in March–August 2020, the literature review covered the articles published between 1 January 2015 and the present. Databases of Scopus and Web of Sciences articles were used for cross-checking. We used a multi-query search strategy. The final results were selected after several search passes by qualitative evaluation of the number of results obtained and their relevance.

Table 1 lists the queries used and the number of articles defined by each of them. The entire bibliography of the included publications was manually checked for compliance with the subject of the search by title and abstract. Articles in the title and/or abstract containing “medical plants COVID” were passed to the full-text selection stage by default. After excluding intersections in all search databases, 318 sources remained under consideration.

## 3. Results and Discussion

Medicinal plants have great potential for use as alternative medicines and are the basis for the discovery of natural compounds for the development of therapeutic agents in pharmacology. Flavonoids of medicinal plants are considered to be powerful immunomodulatory agents [14]. For colds, doctors in Russia traditionally advise to use herbs and forest berries, such as raspberries, rosehip, sage, chamomile, St. John’s wort, etc. They can be used for brewing teas and making gargle solutions. However, the new coronavirus infection is more insidious than all previously encountered flu infections. It is known that the lungs are the organ most severely affected by COVID-19, similar to SARS [15,16]. Thus, special attention was paid to medicinal plants that protect the lungs and support the immune system.

Traditionally, ginger and ginger volatile oils, curcumin, *Panax* L. (*Araliaceae*) and garlic are recommended and used to strengthen immunity and reduce the likelihood of inflammatory respiratory diseases [2,17,18]. In an in vitro study [19], *Allium sativum* L. (*Amaryllidaceae*) extract inhibited influenza A (H_1_N_1_) virus by inhibiting the synthesis of viral nucleoproteins and polymerase activity. The paper recommended a decoction of *Allium cepa* L. (*Amaryllidaceae*) for colds [20].

Based on the experience gained during the SARS epidemic, we selected plants that are effective before or at the initial stage of infection. Roy et al. suggested a number of medicinal plants with these properties, although most of them grow in warm and hot areas, but there are plants found in Northeast Asia [11].

The action of *Lycoris radiate*, *Artemisia annua* and *Pyrrosia lingua* against severe acute respiratory syndrome (SARS) is noted, which is determined by the active substances of these plants and has been confirmed in COVID-19 therapeutic practices [21,22].

*Lycoris radiata* (L’Hér.) Herb. (*Amaryllidaceae*) originally grew in China, Korea and Nepal and was later distributed to other countries, including Russia. It is a source of pharmacologically active alkaloids, with lycorine being the main one [11,23,24,25,26]. Lycorine has selective antiviral activity, including against severe acute respiratory syndrome associated with coronavirus and influenza virus [11,21,27].

Wormwood *(Artemisia annua* L. (*Compositae*)) is an annual herbaceous plant that is common in Europe, Asia and North America and is considered a weed in Russia. The plant contains essential oils, ascorbic acid and traces of alkaloids. Wormwood is traditionally used to treat malaria in the form of tea or pressed juice throughout Asia and Africa [28]. It is known that sesquiterpene lactone artemisinin, isolated from wormwood, is the main ingredient of malaria medicines [29]. Modern phytochemical studies have shown that wormwood contains many active components other than artemisinin, including flavonoids, coumarins, other sesquiterpenes, volatile oils, monoterpenoids and lignans, which determine the prospects for its use in preventive measures [30,31,32]. The antitumor [33], antioxidant [34], anti-inflammatory [35] and anti-asthmatic [36] activity of these wormwood components has already been proven.

*Pyrrosia lingua* (Thunb.) Farw. (*Polypodiaceae*) is a perennial plant that grows in China, India, Japan and other territories of the Asia-Pacific region, and in Russia, it is found in the Far East. Plants of this type contain chlorogenic acid, flavonoids and xanthones. It is known (in vitro study) that flavonoids contribute to antioxidant capacity more than vitamin C and other antioxidants [37,38,39].

The components, including phenolic compounds, of *Isatis indigotica* Fortune ex Lindl. are described as natural with activity against SARS-CoV [22,40,41]. *Isatis indigotica* is widely used in traditional Chinese medicine for the treatment of influenza, viral pneumonia and hepatitis [42,43]. It grows in Armenia, Lebanon, Turkey, Kazakhstan, China, many European countries, North Africa and Russia (North Caucasus, Eastern Siberia and the European region). For centuries, the Chinese have used the roots of *Isatis indigotica* in the clinical treatment of flu, colds, fevers, hepatitis and encephalitis [43,44]. The main chemical components of the plant are alkaloids, flavonoids and phenolic acids [45,46,47,48], which determine its antibacterial, antitumor, anti-inflammatory, antiviral and antioxidant activity [43,49,50,51,52]. It was found that this plant’s polysaccharides also exhibit antiviral activity against herpes simplex virus type II [53], influenza A virus (IAV) [48] and strengthen the immune response in mice as part of the flu vaccine [54].

Liquid elderberry extract is characterized by antiviral action in vitro against influenza, as well as respiratory bacterial pathogens [22,55]. There is preclinical evidence that elderberry (*Sambucus nigra* L. (*Adoxaceae*)) inhibits the replication and viral attachment of the human coronavirus NL63 (HCoV-NL63) [56], which differs from COVID-19 but also belongs to coronaviruses. Elderberry is the most effective means of preventing or combating coronavirus infections at an early stage [57]. The authors of [58] provide evidence of the effectiveness of elderberry use against influenza, which can be useful in determining COVID-19 preventive tactics (the authors provide daily recommendations for a typical daily dosage of elderberry extract for adults and children).

There is some antiviral activity of components of dandelion water extract, which prevents infection and reduces the growth of virus cells of influenza type A and H_1_N_1_ [22,59]. *Taraxacum officinale* L. Weber ex F.H.Wigg (*Compositae*) is one of the most common plants in Ukraine, Belarus, the Caucasus, Moldova, Transnistria, Central Asia and Russia, including Siberia and the Far East. It is often considered as a weed in fields and gardens. Milky juice of the plant contains taraxacin and taraxacerin, 2–3% rubber substances, while blossoms and dandelion leaves are rich in taraxasterol, flavoxanthin, vitamins (C, A, B2, E), choline and saponines, and roots are rich in triterpene compounds (taraxasterol, taraxerol, pseudotranslation, β-amyrin) and sterols (β-sitosterol, stigmasterol, tarksol) [60,61].

Spirooliganone from the roots of *Illicium oligandrum* Merr. & Chun (*Schisandraceae*), chalconoids from *Glycyrrhiza inflata* Batalin (*Leguminosae*) [22,62], xanthones from *Polygala karensium* Kurz. (*Polygalaceae*) [22,63] and *homosoflavonoids* from *Caesalpinia sappan* L. (*Leguminosae*) [22,64] showed powerful antiviral activity [65,66], determining the prospects of using secondary plant metabolites both for inhibiting the growth of influenza cells and possibly coronavirus infection, and for therapeutic purposes.

The antioxidant and antiviral properties of plant-derived flavonoids are described in [2,67,68]. Quercetin inhibits the penetration of SARS-CoV into host cells and shows antiviral activity against HIV-luc/SARS [69,70,71]. The elixir approved by the FDA in the USA for use in SARS-CoV-2 therapy contains quercetin. Quercetin is widely present in leafy vegetables, red onions, seeds and grains, in medicinal plants, is a plant flavonol from the group of flavonoid polyphenols and has proven itself as a pharmacological agent in the treatment of inflammation and oncology [72]. Flavonoid baicalin is isolated from *Scutellaria baicalensis* Georgi (*Lamiaceae*) and is used in traditional Chinese herbal medicine. It proved its antiviral activity against 10 clinical isolates of ARVI using neutralizing tests [73].

*Saposhnikovia divaricata* (Turcz.) Schischk. (family *Apiaceae*) is found in the Northern and Northeastern territories of China, cultivated in many other provinces. As a traditional Chinese medicine, dried root and/or its ethanolic extract are used. It is also used in medical practice in Japan [74]. In the Russian Federation, *Saposhnikovia divaricata* grows in Eastern Siberia and the Far East. The anti-inflammatory, analgesic, antipyretic, anti-allergic, antioxidant, antitumor and antiviral activity of its root extracts has been established [75,76,77,78]. Chromones, coumarins, unsaturated fatty acids, volatile oils and polyacetylene compounds were isolated from the chemical composition [74,79,80]. Polysaccharides of the dried root of *Saposhnikovia divaricata* possess immunoregulatory and antitumor characteristics [77].

*Hyssopus officinalis* L. (*Lamiaceae*) grows in large numbers on dry, stony, calcareous soils in Europe and Southwest and Central Asia. On the territory of Russia, it is found only in the Far East [81,82]. Health-preserving properties of hyssop are due to the richness and variety of secondary metabolites of their extracts [82]. Leaf extracts and essential oils of *Hyssopus officinalis* have been widely studied, both chemically and biologically. Its antimicrobial, antioxidant, antifungal, insecticidal and antiviral effects have been studied [82,83,84,85]. The antibacterial and antifungal properties of hyssop have been attributed to the presence of pinocamphone, isopinocamphone and β-pinene. The antiviral activity of the plant is explained by the presence of caffeic acid, tannins and unidentified high-molecular compounds [85,86].

Golden root (*Rhodiola rosea* L.) belongs to the plant family *Crassulaceae* and is considered a plant adaptogen with various protective effects (antidiabetic, anticancer, anti-aging, cardio-protective and neuroprotective). It is known that golden root has an excellent immunoregulatory effect and weakens inflammatory damage in various diseases by regulating the differentiation of immune cells, activating inflammatory signaling pathways and secreting inflammatory factors [87,88,89]. Polysaccharides isolated from the rhizomes and roots of *Rhodiola rosea* have antioxidant, antiviral and antitumor activity and are widely used in traditional Chinese medicine [90,91,92]. Active compounds (salidroside, tyrosol, ferulic acid, kaempferol, Gallic acid, catechin and phenethyl ether of caffeic acid, etc.) have confirmed anti-inflammatory effects in various models in vivo and in vitro [89,93,94], including induced lung damage in mouse models [91,95]. It was found that salidroside can weaken asthma [96].

Rhaponticum *(Rhaponticum carthamoides* (Willd.) Iljin. (*Compositae*)) is a perennial herb that grows in the Altai Mountains, Western and Eastern Siberia and Central Asia. In medicine, rhizomes with rhaponticum roots are used as a general tonic and adaptogenic drug [97,98,99]. It is widely used as a dietary supplement and is valued as a rich natural source of ecdysteroids, which are present in all parts of the plant [97]. Studies of the phytochemical composition have shown that the plant contains various classes of secondary metabolites (phenolic acids with caffeoylquinic acid derivatives, flavonoids, ecdysteroids, polyacetylenes, sesquiterpene lactones and triterpenoid glycosides) [97,100,101]. Extracts have a wide range of biological activity due to the presence of flavonoids, sesquiterpene lactones and polyacetylenes [98].

Amur maackia *(Maackia amurensis* Rupr. (*Leguminosae*)) is common in Korea, Japan, Northeastern China and Eastern Russia. The trunk bark of this tree has traditionally been used for the treatment of cancer, cholecystitis, arthritis and hyperthyroidism, as well as for hepatoprotection [102,103]. The chemical composition of *M. amurensis* has been determined with identified isoflavonoids, polyphenol and isoflavone glycosides, prenylated flavonoids [104,105,106]. Flavonoids are known for their antioxidant and antiviral activity [107], which allows this plant to be included in the group of plant prevention agents during the coronavirus pandemic.

Licorice (*Glycyrrhiza inflata* Batalin.) grows in Europe, North Africa, Asia, Russia, Siberia and the Caucasus. For centuries, dried roots and rhizomes of glycyrrhiza (*Leguminosae*) have been used as herbal medicines in many countries. *Glycyrrhiza glabra* L. is cultivated in Europe; *Glycyrrhiza uralensis* Fisch. and *Glycyrrhiza inflata* are common in traditional Chinese medicine. Licorice extracts have antiviral, antimicrobial, antioxidant, anti-inflammatory and antitumor characteristics [108,109], determined by triterpene saponins and flavonoids. The main triterpene saponin is glycyrrhizin (5–10% of roots), from phenolic compounds liquirytigenin, isolivytigenin and their glycosidic derivatives (around 1% of licorice water extract) [110,111,112].

The authors of [113] describe the results of studying the antiviral characteristics (against avian influenza - H_5_N_1_) of extracts of five Asian medicinal plants (*Andrographis paniculata*, *Curcuma longa*, *Gynostemma pentaphyllum, Kaempferia parviflora, Psidium guajava*). Both water and ethanol extracts from all the plants studied showed significant antiviral activity against H_5_N_1_ virus, but the authors name *C. longa* and *K. parviflora* the most active ones.

*Andrographis paniculata* (Burm.f.) Nees (*Acanthaceae*) is common in tropical Asian countries, often in isolated locations. Wild populations can be found throughout Southern India and Sri Lanka; cultivated populations are found in Northern India, Java, Malaysia, Indonesia, the West Indies, parts of the Americas, the Philippines, Hong Kong, Thailand, Brunei, Singapore and other regions of Asia. The main compound of the plant is andrographolide secondary metabolite, the highest content of which is concentrated in the stem, flowering tops and roots [114]. Antitumor, anti-inflammatory, antimicrobial, antioxidant, cytotoxic, hepatoprotective, immunostimulating and antiviral activities of this metabolite have been established [114,115]. The ability of *Andrographis paniculata* to treat viral respiratory infections has been confirmed in ayurvedic and other non-traditional medical practices [116,117]. The components of this plant inhibit the increased NOD-like receptor protein (NLRP3), caspase-1 and interleukin-1β molecules that are actively involved in the pathogenesis of SARS-COV, which determines the potential in the treatment and prevention of SARS-CoV-2 [118].

*Curcuma longa* L. (*Zingiberaceae*) is a perennial herbaceous plant of the Ginger family found wild in India and South Asia, cultivated in areas with warm climates, including the countries of Northeast Asia. The plant’s roots have antiviral, antimicrobial, immunomodulatory, anti-inflammatory and other characteristics [119]. The main active ingredient of the plant is polyphenol curcumin, which also contains lipids, dietary fiber, carbohydrates, vitamins, minerals, polyunsaturated fatty acids and essential oils. Curcumin is well known for its anti-inflammatory potential; many clinical studies have been conducted to evaluate its bioactive effect in various inflammatory conditions. Curcumin has a relieving and preventive effect on respiratory disorders [120].

*Gynostemma pentaphyllum* (Thunb.) Makino (*Cucurbitaceae*) is found in China and in South and East Asia. Traditional medicine uses it in the form of teas, extracts and dietary supplements. It is widely used in Chinese alternative medicine. Positioned as a general tonic, it also treats viral and infectious diseases [121,122,123,124]. Due to the significant content of saponins and antioxidants, it has a rejuvenating effect [125]. In terms of the saponin content, *Gynostemma pentaphyllum* significantly outperforms ginseng. Due to this, the plant effectively increases physical endurance and tones up the body. Rich in vitamins, the plant prevents colds and viral diseases. Amino acids provide better protein digestibility.

*Kaempferia parviflora* Wall. ex Baker *(Zingiberaceae*) is an herbaceous plant native to Thailand. It has strengthening and stimulating properties. Due to the high content of methoxyflavones, this plant demonstrates antioxidant properties in vitro. Methoxyflavones (flavones with an attached methoxyl group) are the main components of the plant (there are three of them: 3,5,7,3′, 4′-pentamethoxyflavone (PMF), 5,7-dimethoxyflavone (DMF), and 5,7,4′-trimethoxyflavone (TMF)), which are most often studied. Kaempferosides—unique components of *Kaempferia parviflora*—are not considered active ingredients. *Kaempferia parviflora* rhizomes have been reported to have antioxidant, anti-inflammatory and antimicrobial activity due to the high content of biologically active phenolic and methoxyflavone compounds [126]. It was found that the chemical components and extracts of *Kaempferia parviflora* have a variety of biological active properties. Flavone (5-hydroxy-7-methoxyflavone and 5,7-dimethoxyflavone) inhibits viral protease, flavonoids inhibit *Mycobacterium tuberculosis* and *Candida albicans*. Rhizomes prepared in the form of alcohol or water decoction are prescribed for the treatment of various diseases [127].

*Psidium guajava* L. (*Myrtaceae*) is found in tropical and some subtropical areas of Asia, Africa and South and North America. In the traditional medicine practiced by many cultures and peoples of these territories, *Psidium guajava* is used for inflammation, diabetes, fever, lung diseases, hypertension, etc. The greatest biological activity is demonstrated by the bark of shoots and unripe fruits; to a greater extent, it is associated with the antioxidant effect of secondary metabolites, mainly flavonoids. The bark contains diglycosides of ellagic acid, ellagic acid, leucodelfinidine and saponins. Iha et al. [128] found tannins (epicatechin) and flavonoids (rutin and quercetin) in the chemical composition of the plant. *Psidium guajava* has antibacterial, antifungal, antiviral, antioxidant and other properties [129,130,131].

*Echinacea purpurea* (L.) Moench (*Compositae*) has a certain potential in the prevention of COVID-19. Native to America, this plant is cultivated everywhere. The ground part of *Echinacea purpurea* contains polysaccharides, essential oils, flavonoids, hydroxycinnamic acids, tannins, saponins, echinacin, echinolone, echinacoside, organic acids, resins and phytosterols; the roots contain inulin, glucose, essential and fatty oils, phenolcarboxylic acids, betaine and resins. All parts of the plant contain enzymes, macro- and microelements. Echinacea is one of the most popular natural health products purchased worldwide, with most commercially available products containing *E. purpurea* alone or mixed with *E. angustifolia* DC. (*Compositae*). Many naturopathic doctors recommend echinacea for immune support [132].

Studies [133,134] have shown that echinacea can reduce the severity and/or duration of acute respiratory infections when taken at the beginning of the disease when the first symptoms appear. *Echinacea purpurea* reduces the risk of recurrent respiratory infections and the incidence of complications. The high content of essential oils, antioxidants, organic acids, vitamins of groups A, B and E. Echinacea-based preparations boosts the immune system and helps the body to fight flu viruses, herpes and SARS. The immunomodulatory, antiviral and anti-inflammatory effects of this plant can serve as a basis for research on its activities in relation to COVID-19.

Effects and activity of the medicinal plants are identified in Table 2.

## 4. Conclusions

Epidemics and pandemics are always a test for humanity, and COVID-19 is no exception. Currently, there is no established pharmacological strategy for the prevention and/or treatment of a new coronavirus infection. Since the beginning of the COVID-19 epidemic, people around the world have been under constant stress and are experiencing significant negative emotions, fear, anxiety and anger [135,136]. All this immediately affects the state of their immune system and reduces the body’s resistance not only to the SARS-CoV virus but to other viral and bacterial infections as well. It is established that believing in the world’s justice helps to increase the level of positivity in a person’s life [137], while family support also plays an important role. In this situation, we only have to wait for the vaccine and strengthen immunity to keep ourselves and our loved ones healthy.

The list of medicinal plants that grow in the eastern territories of our country and can help support the body during the pandemic is yet to be completed. We are aware of the danger of unauthorized use of medications without a doctor’s supervision. There can be only one answer: strictly follow the recommendations of pharmacists and stop immediately in the event of the slightest complications or inadequate reactions of the body.

It is believed that the only sustainable way to survive in the current situation is to boost the immune system. Many foods and herbs have antiviral and immunomodulatory properties. A balanced diet and dietary intake of nutrients affect the immune system through gene expression, cell activation and modification of signaling molecules. In addition, various food ingredients are determinants of the gut microbial composition and subsequently form immune responses in the body [138,139,140]. A diet combined with medicinal plants with immunomodulatory, antiviral and anti-inflammatory effects can significantly enhance this protection.

Diet plays a huge role in disease prevention. During seasonal colds, it should be rich in vitamins and antioxidants. Key dietary components, such as vitamins C, D, E, zinc, selenium and polyunsaturated fatty acids, have an immunomodulatory effect. Recent studies have shown that dietary supplements can have beneficial effects, potentially decreasing the viral load of SARS-CoV-2 and reducing the recovery period in patients with COVID-19 [107,141].

Vitamin A supplementation reduces the incidence of various infectious diseases and lowers the mortality rate [107,142]. Vitamin B2 together with UV light effectively reduces the SARS-CoV titer in human plasma products [143]. Vitamin D deficiency in calves leads to greater infection with bovine coronavirus [144]. Vitamin C is widely known both as an antioxidant and as an aging process inhibitor [145]. It also participates in chemotaxis and phagocytosis, increasing the amount of reactive oxygen species for the destruction of microorganisms. It is often considered as an antibacterial agent and has an anti-inflammatory effect [146]. It was found that vitamin C inhibits lung fibrosis [59,107], has a direct virulent effect at higher concentrations and reduces the viral load on infected cells with Ebstein–Barr virus [2,147]. However, there is still no consensus on the antiviral effectiveness of this vitamin. However, it is vitamin C infusion that has already been tested on patients with pneumonia infected with SARS-CoV-2, and numerous clinical studies are continuing with their relevance maintained [2]. Products rich in vitamin C traditionally include lemons, oranges, kiwis, guavas and grapes, but it is extremely difficult to cultivate them in Siberia and the Far East. However, nature is also rich in fruits with this vitamin: black currant (*Ribes nigrum* L. (*Grossulariaceae*)), sea buckthorn (*Hippophae rhamnoides* L. (*Elaeagnaceae*)), rosehip (*family Rosaceae*), and vegetable crops such as broccoli (*Brassica oleracea* var. *italica* Plenck (*Brassicaceae*)), cauliflower (*Brassica oleracea* var. *botrytis* L. (family *Brassicaceae*)), sweet pepper (*Capsicum annuum* L. (*Solanaceae*)), wild garlic (*Allium ochotense* Prokh. (*Amaryllidaceae*)), etc. Moreover, in every territory, perhaps with the exception of the Arctic and Antarctic, there are plants that can improve health, while emotional state [148,149] plays an important role in all these activities too.

## Figures and Tables

**Table 1:** Strategies for finding literary sources of the review.

Data Base	Search Query (Title/Abstract/Keywords)	Number of Articles	Matching the Search
Scopus	Medical Plants COVID	18	4
	Strengthen immunity COVID	4	2
	Medical Plants Immunity	110	22
	Medical Plants influenza	61	18
	Antiviral Medical Plants	215	36
WoS	Medical Plants COVID	2	1
	Strengthen immunity COVID	8	2
	Medical Plants Immunity	32	3
	Medical Plants influenza	17	9
	Antiviral Medical Plants	70	12

**Table 2:** Effects of medicinal plants.

Plant	Condition	Solvent	Part Used	Effects	References
*Allium cepa*	Extract	Chloroform	Bulb	Antiviral activity IFA H_1_N_1_)	[19]
	Natural	-	Bulb	Antiviral activity (SARS—cold and flu)	[20]
*Allium sativum*	Extract	Ethanol or aqueous	Roots	Antiviral activity (IFA—H_1_N_1_)	[19]
	Natural	-	Bulb	Immunoregulatory effect	[20]
*Andrographis paniculata*	Extracts	Aqueous or ethanol	Leaves	Antiviral activity (IF H_5_N_1_)	[113,116]
*Artemisia annua*	Extract	Ethanol	Whole plant	Antiviral activity (SARS-CoV)	[21,22]
*Caesalpinia sappan*	Extract	Ethanol	Heartwood	Antiviral activity (IFA—H_1_N_1_, H_3_N_2_, H_9_N_2_)	[22,64]
*Curcuma longa*	Extract	Aqueous or ethanol	Roots	Antiviral activity (IF H_5_N_1_), prevention	[113,120]
*Echinacea purpurea*	Essential oil	-	Flowers	Antiviral activity (SARS), Immunoregulatory, anti-inflammatory effects	[132,133,134]
	Syrup	-	Flowers, Roots		
	Extract	Ethanol	Flowers		
	Sap	-	Herb		
	Herb mix	-	Herb and root		
*Glycyrrhiza glabra*	Powder	-	Centuries, dried roots and rhizomes	Antiviral, antimicrobial, antioxidant, antitumor activity	[108,109]
*Glycyrrhiza inflata*	Extract	Acetone	Roots	Antiviral activity (IFA—H_1_N_1_)	[22,62]
	Powder	-	Centuries, dried roots and rhizomes	Antiviral, antimicrobial, antioxidant, antitumor activity	[108,109]
*Glycyrrhiza uralensis*	Powder	-	Centuries, dried roots and rhizomes	Antiviral, antimicrobial, antioxidant, antitumor activity	[108,109]
*Gynostemma pentaphyllum,*	Extract	Aqueous or ethanol	Leaves or ground part	Antiviral (IF H_5_N_1_), antioxidant, antiproliferative activity	[113,123,124]
*Hyssopus officinalis*	Extract	Ethanol	Leaf	Antiviral, antimicrobial, antioxidant, antifungal, insecticidal activity	[84]
	Essential oils	-	Leaf, flower and stem		[85,86]
*Illicium oligandrum*	Powder	-	Roots	Antiviral activity (IFA)	[22,65]
*Isatis indigotica*	Extract	-	Roots	Antiviral activity (SARS-CoV, influenza—H_1_N_1_, H_3_N_2_, H_6_N_2_, H_9_N_2_, viral pneumonia, and hepatitis)	[22,48]
*Kaempferia parviflora,*	Extract	Aqueous or ethanol	Roots	Antiviral (IF H_5_N_1_) and antimicrobial activity	[113,126,127]
	Essential oil	-			
*Lycoris radiate*	Extract	Ethanol	Stem cortex	Antiviral activity (SARS-CoV)	[21,22]
*Maackia amurensis*	Extract	Ethanol	Bark	Antiviral and antioxidant activity	[105,106,107]
*Polygala karensium*	Extract	Ethanol	Roots	Antiviral activity (IFA)	[22,63]
*Psidium guajava*	Extract	Aqueous or ethanol	Leaves	Antiviral (IF H_5_N_1_), antimicrobial activity	[113,128,129,131]
		Ethanol	Whole plant		
		CH_3_OH/H_2_O/formic acid	Pulp	Antioxidant activity	[130]
*Pyrrosia lingua*	Extract	Chloroform	Leaf	Antiviral activity (SARS-CoV)	[21,22]
*Rhaponticum carthamoides*	Essential oil	-	Roots	antiviral, antimicrobial, antioxidant and antitumor activity, immunoregulatory activity	[98]
	Extract	Acetone, ethyl acetate or methanol	Leaf		[97]
*Rhodiola rosea*	Extract	Aqueous	Roots	Emmunoregulatory effect; antiviral, antioxidant, anti-asthma activity	[92,94,96]
*Sambucus nigra*	Extract *	-	-	Antiviral activity (IFA, IFB)	[22,55]
*Saposhnikovia divaricata*	Extract	Ethanol	Roots	Emmunoregulatory effect	[77]
*Taraxacum officinale*	Extract	Aqueous	Herb	Antiviral activity (IFA—H_1_N_1_)	[22,59]

IF—influenza, IFA—influenza A, IFB—influenza B, SARS—severe acute respiratory syndrome, SARS-CoV—viral respiratory disease of zoonotic origin. * commercial preparat Rubini, BerryPharma AG, Germany.
